# Estimating the Clinical and Economic Impact of Switching from the 13-Valent Pneumococcal Conjugate Vaccine (PCV13) to the 10-Valent Pneumococcal Conjugate Vaccine (PCV10) in Italy

**DOI:** 10.3390/pathogens9020076

**Published:** 2020-01-22

**Authors:** Filippo Ansaldi, Sarah Pugh, Daniela Amicizia, Roberto Di Virgilio, Cecilia Trucchi, Andrea Orsi, Alessandro Zollo, Giancarlo Icardi

**Affiliations:** 1 Department of Health Sciences, University of Genoa, 16132 Genoa, Italy; daniela.amicizia@unige.it (D.A.); andrea.orsi@unige.it (A.O.); icardi@unige.it (G.I.); 2 IRCCS San Martino University Hospital, 16132 Genoa, Italy; cecilia.trucchi@regione.liguria.it; 3 Liguria Health Authority, A.Li.Sa., 16121 Genoa, Italy; 4 CIRI-IT, Centre of Influenza and other Respiratory Infections, 16132 Genoa, Italy; 5 Pfizer, Collegeville, PA 19426, USA; Sarah.Pugh@pfizer.com; 6 Pfizer, 00188 Rome, Italy; Roberto.Divirgilio@pfizer.com (R.D.V.); Alessandro.Zollo@pfizer.com (A.Z.)

**Keywords:** cost-effectiveness analysis, impact, pneumococcal infections, pneumococcal vaccines

## Abstract

**Background**: Invasive and non-invasive pneumococcal diseases are significant health and economic burdens, especially in children and the elderly. Italy included the 7-valent (PCV7) and 13-valent pneumococcal conjugate vaccine (PCV13) in the National Immunization Program in 2007 and 2010, respectively, allowing a dramatic reduction in the burden of pneumococcal disease. In the era of budget constraints, decision-makers may consider switching from the higher-valent, more costly PCV13, to the lower-cost PCV10. This study estimated the potential public health and economic impact of changing vaccine programs from PCV13 to PCV10 in Italy. **Methods**: A decision-analytic forecasting model estimated the impact of PCV programs. Real-world surveillance data were used to forecast serotype distribution and disease incidence among children and the elderly over a specified 5-year time horizon. Costs and outcomes included estimates of cases and deaths avoided, quality-adjusted life years (QALYs) gained, and total costs from a payer perspective, discounted at an assumed rate of 3.0%, and robustness validated through several scenarios and sensitivity analyses. **Results**: A switch from PCV13 to PCV10 would increase invasive pneumococcal disease (IPD) cases by 59.3% (4317 cases) over a 5-year horizon, primarily due to serotypes 3 and 19A. Pneumonia increased by 8.3% and acute otitis media (AOM) by 96.1%. Maintaining a PCV13 program would prevent a total incremental 531,435 disease cases (1.02M over a 10-year time horizon) and 641 deaths due to invasive pneumococcal disease (IPD), with €23,642 per QALY gained over 5 years versus PCV10. One-way and probabilistic sensitivity analyses showed that a PCV13-based program remained cost-effective in 99.7% of the simulations in Italy as parameters varied within their plausible range; percent vaccinated had the most impact. **Conclusions**: Maintaining the PCV13 strategy would provide substantial public health and economic benefits in Italy and is cost-effective. Switching from PCV13 to PCV10 would increase the incidence of pneumococcal disease primarily linked to re-emergence of serotypes 3 and 19A.

## 1. Introduction

*Streptococcus pneumoniae* (SP) is associated with significant morbidity and mortality in children, adults, and older adults, but the pneumococcal burden dramatically decreased following vaccine implementation [1]. There are over 90 distinctive serotypes of this bacterium which vary in geographical prevalence, according to age group, vaccination pressure, surveillance methods, environments, i.e., antibiotic use, and host factors [2]. Furthermore, natural variation, at least partly due to population immunity, induced by circulating pneumococcal strains, may play a significant role in determining the epidemiological picture.

The first pneumococcal vaccine, Pneumovax (PPSV23), a polysaccharide vaccine against 23 prevalent serotypes of SP was introduced in the United States in 1983, and there is strong evidence of its ability to reduce invasive disease in adults [3,4,5,6]. PPSV23 showed many shortcomings, including inconclusive evidence of protection against non-invasive disease, poor immunogenicity in children, especially those under the age of two years, and a rapid decrease of effectiveness with time since administration. To overcome these limitations, pneumococcal vaccines containing polysaccharide covalently conjugated to carrier protein were introduced: in 2000 and in 2010, 7-valent pneumococcal (PCV7) and 13-valent (PCV13) conjugate vaccines, respectively, were licensed in the United States. The carrier protein used for both vaccines was a non-toxic mutant of diphtheria toxin (CRM 197). Protein D, derived from non-typeable *Haemophilus influenzae*, tetanus, and diphtheria toxoids were used for the 10-valent pneumococcal conjugate vaccine (PCV10) that was authorized in Europe in 2009.

In Italy, PCV7 was introduced to the private market in 2002, followed by a universal childhood vaccination program against SP in some administrative regions, i.e., Liguria in May 2003. In 2007, PCV7 was implemented as part of a national immunization program and replaced with PCV13 in May 2010, according to the Ministry of Health recommendation [7]. The 2012–2014 National Vaccination Plan included the recommendation for 3 PCV13 doses for newborns at 3, 5–6, and 11–13 months of age. As of 2013, PCV vaccination coverage in children aged 24 months progressively increased from the 2005 to 2009 birth cohort, with considerable variability between the regions, from 44.7% to 98.5% in 2011 [8]. The latest available data reports national coverage is now higher than 90% among children aged two years belonging to 2015 birth cohort [9].

As far as vaccination implementation in adults, since 2005, the Italian National Vaccination Plan has recommended the administration of PPSV23 to subjects of any age who present high-risk conditions or comorbidities. The last available edition of the Italian Vaccination Plan (2017–2019) [10], reported that PCV13, followed by a dose of PPSV23 at least 8 weeks later, was indicated for high-risk adolescent and adults and subjects aged 65 years and older. Nevertheless, several years previously, following approval for PCV13 use among adults aged >50 years to prevent pneumonia and invasive disease caused by SP, several Italian regions implemented age-based pneumococcal immunization programs in adults and the elderly, recommending a single dose of PCV13 or a dose of PCV13 followed by a dose of PPSV23 [11,12].

Even though pneumococcal immunization programs have been proven to reduce the burden of invasive and non-invasive disease, they represent a significant budget impact on the health system. To optimize health resource utilization, vaccine program funding bodies continuously look for ways to reduce healthcare expenditure. Several countries, including Belgium, New Zealand, Morocco, El Salvador, Quebec, and some regions in Sweden and Italy switched childhood pneumococcal vaccination programs from PCV13 to PCV10 for economic reasons. For example, in Belgium, PCV10 replaced PCV13 in the northern part of the country (Flanders) in July 2015 and in the southern part (Wallonia) in May 2016.

Interpreting the epidemiological impact of the switch in childhood pneumococcal vaccination programs from PCV13 to PCV10 is the center of a heated debate in the scientific community [13,14] as Belgium has observed an increase in disease, primarily due to serotype 19A. Uncertainties about serotype replacement, herd effects, serotype-cross protection, and country-specific serotype prevalence make estimates of the clinical and economic impact of switching from PCV13 to PCV10 in a childhood pneumococcal vaccination program difficult to generalize, creating a challenge for public health strategy. The existing evidence on the clinical and economic impact of a PCVs program specific to Italy may be limited by the use of effectiveness data from countries with a possibly different epidemiology than Italy.

Therefore, the aim of this study was to expand upon this literature by using a decision-analytic forecasting model and real-world data from Italy to estimate the potential public health and economic impact of changing vaccine programs from a higher (PCV13) to lower (PCV10) valent vaccine in comparison with maintaining the current vaccination strategy of PCV13 in Italy.

## 2. Results

The clinical impact of switching from the PCV13 to PCV10 forecasted over 5 years and the current serotype distribution are first presented for children ≤2-years-old and adults ≥65 years, since these populations are the most susceptible to pneumococcal disease and form a large proportion of the total burden. Incremental cases, deaths, incremental costs, and Quality Adjusted Life Years (QALYs) under a PCV13 versus PCV10 vaccination program, over a 5 year time horizon were reported for all ages. Incremental values were calculated as the difference between estimated cases, deaths, and costs under a PCV13 vaccine program and estimated values under a PCV10 vaccination program.

### 2.1. Epidemiological Results

**Figure 1:**
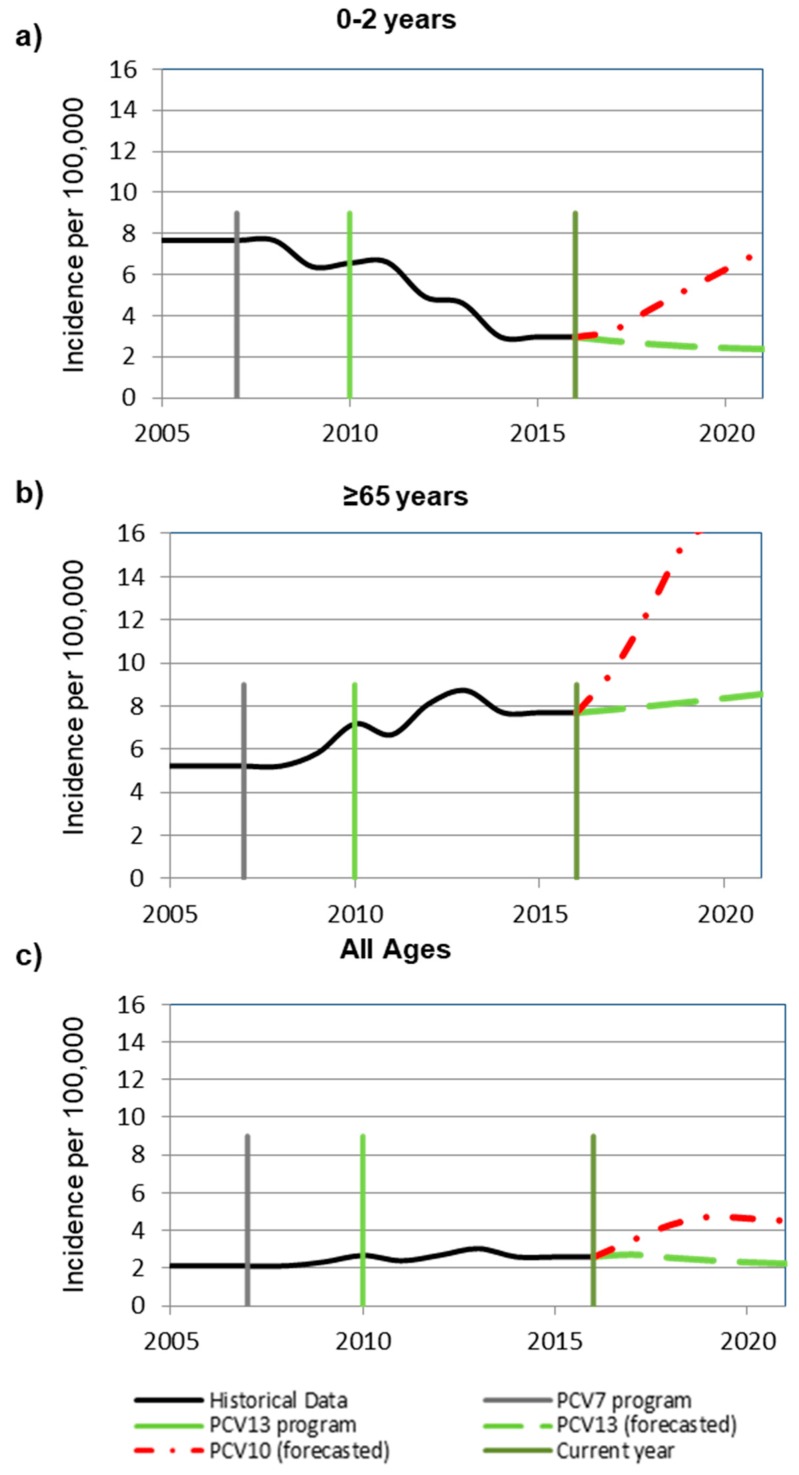
Forecasted invasive pneumococcal disease incidence trends based on historical invasive pneumococcal disease rates in (**a**) 0 to 2-year-olds (**b**) ≥65-year-olds, and (**c**) all ages. Historical data, 13-valent pneumococcal conjugate vaccine (PCV13) program, 7-valent (PCV7) program, 10-valent (PCV10) forecasted, 13-valent (PCV13) forecasted, and current year reflecting the “year of modeled switch” are considered.

**Figure 2:**
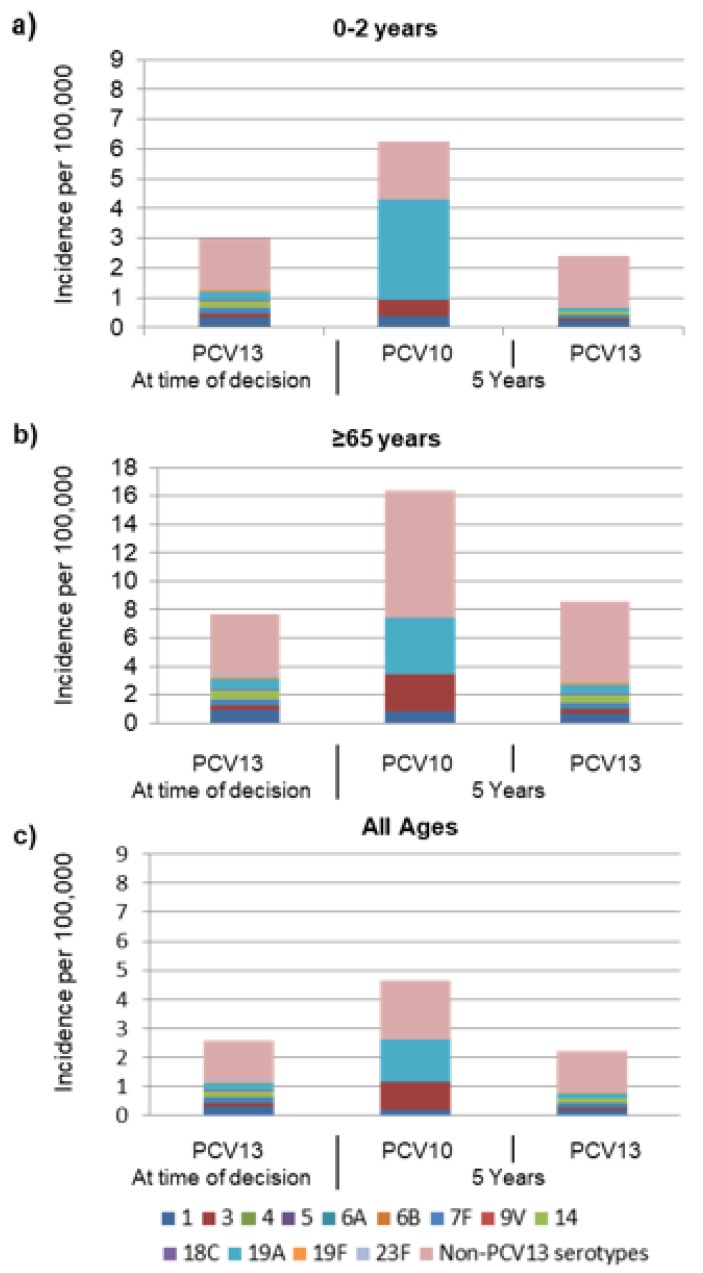
Invasive pneumococcal disease serotype distribution currently and forecasted at 5 years under either PCV10 or PCV13 in (**a**) 0 to 2-year-olds (**b**) ≥65-year-olds, and (**c**) all ages. “At time of decision” indicates the “current state of pneumococcal disease with the PCV13 strategy”.

Based on epidemiological assumptions, at the year of modelled switch, the incidence of invasive pneumococcal disease (IPD) among 0–2-year-olds was 2.98 per 100,000 with 55% of remaining disease due to non-PCV13 type disease (Figure 1a). In children 0–2 years old, after a switch from PCV13 to PCV10, IPD was estimated to increase from 2.98 to 6.2 cases per 100,000 over 5 years (Figure 1 and Figure 2). Estimated increases in disease were primarily due to serotype 3, increasing from 0 to 0.56 per 100,000, and serotype 19A, increasing from 0 to 3.4 per 100,000 and comprising 65% of the estimated disease (Figure 2). In contrast, when maintaining PCV13, disease was estimated to remain stable at a low incidence of 2.5 per 100,000 with most residual disease caused by non-PCV13 type disease.

In adults aged 65 years and older, after a switch, IPD was predicted to increase from 7.7 to 16.4 cases per 100,000, with the majority of disease caused by non-PCV13 serotypes (9.0/100,000) and serotypes 3 (2.7/100,000) and 19A (3.9/100,000) (Figure 2). A slight increase in incidence (from 7.7/100,000 to 8.4/100,000) was estimated when maintenance of PCV13 was modelled due to non-PCV13 type disease.

The steep trend for >65-year-olds may be explained by the current serotype distribution in Italy and the trends observed in Finland (the country from which PCV10 trends were applied) (Figure 1b). Currently in Italy, a large proportion of remaining disease is due to non-vaccine type disease. In Finland, under a PCV10 program non-vaccine type disease has been increasing.

Therefore, when we apply the PCV10 trend lines from Finland to Italy, there is a steep incline in non-vaccine type disease.

A similar trend was observed when assessed across all ages in which a switch to PCV10 was estimated to result in a 79% increase in IPD to an average of 4.6 cases per 100,000, a relevant increase in incidence of IPD due to serotypes 3 (from 0.1/100,000 to 0.9/100,000) and 19A (from 0.2/100,000 to 1.4/100,000). Across all ages, 2.6 cases of IPD per 100,000 remained with 53% due to non-PCV13 type disease and 8%–10% each due to serotypes 1 and 14 (Figure 1c) [15]. 

### 2.2. Base Case Cost-Effectiveness Analysis Results 

In the base case scenario, a national switch to PCV10 over the next 5 years (2017–2021) was estimated to increase IPD cases by 77% (5491 cases), pneumonia by 10% (41,639 cases), and acute otitis media (AOM) by 71% (393,902 cases), compared with maintaining PCV13 (Table 1). Maintaining a PCV13 program was estimated to prevent 814 deaths due to IPD, compared with switching to PCV10. The total incremental cases avoided with a PCV13 program for both invasive and non-invasive disease were 851,254 and they doubled over a 10 year time horizon (2.1M incremental cases avoided) (data not shown).

By region, the number of cases and deaths prevented over 5 years was estimated relative to population size (Supplementary Table S1). More populous regions such as Lombardia were estimated to incur an additional 70,585 cases and 135 deaths due to pneumococcal disease under a switch in vaccination programs from PCV13 to PCV10 (Supplementary Table S1).

Due to the sustained clinical benefit, maintaining PCV13 was estimated as the more cost-effective intervention at €28,963 per QALY gained over 5 years, compared with PCV10. 

### 2.3. Sensitivity Results

Across four one-way scenarios (Table 2), PCV13 remained cost-effective in all scenarios except when using the Netherlands trend lines for PCV10, though PCV13 was still dominant to PCV10. The one-way sensitivity analysis (Supplementary Figure S1) indicated that a PCV13-based program remained cost-effective in Italy as parameters varied within their plausible range, with the percent vaccinated having the most impact. The probabilistic sensitivity analysis (Supplementary Figure S2) showed that a PCV13-based program remained cost-effective in 99.7% of the simulations and cost-saving in 0.3% of simulations.

## 3. Discussion

In the current analysis, we applied a forecasting framework that leveraged >10 years of PCV experience to predict future serotype dynamics, the incidence of invasive and non-invasive pneumococcal diseases and direct medical costs, comparing the current vaccination strategies with a policy shifting to a lower-valent vaccine in Italian infant vaccination programs.

Although some other studies describe the health and economic impact of switching the infant vaccination program from 13- to 10-valent PCV [16,17,18], this analysis estimates the impact based on the epidemiology and serotype dynamics specific to PCV experience in Italy. In order to better define the serotype dynamics, data from the most sensitive regional surveillance systems were used and were considered representative of the incidence rate across the entire country, as no potential bias was knowingly introduced.

Furthermore, Italy presents a demographic structure characterized by very high aging indices that anticipates that of other European countries and may represent the projected figure for the future.

This study highlights the benefit offered by maintaining PCV13 in comparison with switching to a PCV10 program, by demonstrating lower invasive and non-invasive disease incidence, QALYs gained, and deaths avoided, all of which suggest a greater public health impact of a higher-valent vaccine. In the event of a switch to PCV10, the estimated increase in disease was primarily due to serotypes 3 and 19A in the under 2-year-old and ≥65-year-old populations. These serotypes have been shown to be associated with complicated disease, multidrug resistance, and the need for longer treatment [19]. Therefore, the results of this clinical and economic model could have important implications for public health and could potentially underestimate the complete value of a program including PCV13. These findings are relevant in light of new Advisory Committee on Immunization Practices (ACIP) guidance for PCV13 based on shared clinical decision-making among adults 65 years or older who do not have an immunocompromising condition and who have not previously received PCV13 should receive a dose of PPSV23 [20].

Our results suggest that maintaining PCV13 would be a cost-effective strategy within a five-year time horizon compared with switching to a lower-valent vaccine in all evaluated scenarios except when using the Netherlands trends line, considering the incremental cost-effectiveness ratio (ICER), World Health Organization (WHO), and National Institute for Health and Care Excellence (NICE) cost-effectiveness thresholds. 

The findings obtained by modeling the clinical and economic impact of pediatric pneumococcal conjugate vaccine programs in Italy are consistent with those of several studies [16,17] that applied a similar forecasting decision-analytic model in the Canadian and Mexican contexts and with the real experience of Belgium (Flanders and Wallonia) where the switch caused a ten-fold increase in the number of 19A pneumococcal disease isolates from 2015 to 2017 [13].

The clinical and economic studies underline that PCV programs continue to provide a significant return on investment and PCV13 could provide the greatest healthcare and economic impact. In both countries, the forecasting decision-analytic model showed that maintaining PCV13 is a cost-effective strategy for the majority of scenarios. Our analysis showed positive incremental costs when PCV13 strategy is compared with a switching policy in the considered scenarios. Any difference in results between countries may be explained by the difference in country epidemiology, serotype dynamics, and cost inputs. In Italy, the current incidence of IPD and outpatient pneumonia is lower than that reported in Mexico and Canada in pediatric age groups and cost for a hospitalized pneumonia in Italy is 21% lower than in Mexico and >3 times lower than in Canada (data not shown). The epidemiological picture of serotypes causing IPD is different among countries at the time of the decision: in the 0–2-year age group, in Mexico, IPD incidence was twice in comparison with that observed in Italy, where the incidence due to all serotypes was equal (3/100,000) to that registered in Mexico due to serotype 3, 6A, 6B, 19A. In adults aged 65 and older the burden of disease is completely different in the two countries, as in Italy the incidence of IPD is more than 10 times higher than in Mexico. The forecasted picture after PCV13-PCV10 switching in the 0–2-year cohort is characterized in Italy by a relevant role played by serotypes 3 and 19A representing 65% out of all IPD cases, while in Mexico non-PCV13 serotypes are predicted to cover 58% of IPD cases. In adults aged 65 years and older, 5 years after PCV13-to-PCV10 switching, models in Italy predict a relevant role played by serotypes 3 and 19A reaching an incidence of 2.7/100,000 and 3.9/100,000, respectively, while in Mexico the IPD incidence due to these serotypes is forecasted to be <0.1/100,000 in this age group.

Considering the Italian context, an economic analysis carried out from the National Health System perspective that considered direct costs and compared PCV10 with PCV13 strategy found that vaccination with PCV10 would be slightly more cost-effectiveness for infants using the same cost of vaccines. The advantage of PCV10 was described as being linked to the major effectiveness in the prevention of non-typeable H. influenza (NTHi) AOM [21]. However, this study reported some relevant limitations such as the use of a static model that is not able to accurately capture the long-term evolution of the epidemiology given by vaccines, the absence of direct comparison data, the use of effectiveness data from countries with a possibly different epidemiology than Italy, and the herd effect in the elderly. Furthermore, the low numbers of IPD (cases are rare) could over- and underestimate the predictive trends. 

The main driver of observed differences between the two high valency vaccines was their effect on AOM, which is not their main clinical indication. Furthermore, the estimate included for the impact of PCV10 on NTHi AOM was based on an investigational 11-valent vaccine, and no evidence to date has supported this point estimate for PCV10, which is a different formulation from the investigational vaccine [22,23].

The strengths of our study are that the analysis incorporates the observed trends in serotypes dynamics in the presence and absence of vaccination pressure and the accurate estimates of potential serotypes replacement in case of vaccine switch by modeling each serotype by age group and within a 5-year time horizon.

Furthermore, our analysis is based on real-world data based on seven Italian regional surveillances; therefore, it provides a realist framework. This is translated into capturing vaccine dynamics such as vaccine waning cross-reactivity and herd protection. This study is further strengthened by the high pediatric vaccine coverage for more than a decade and a well-defined serotype picture [24,25].

Like all pharmacoeconomic analyses, the present study has limitations because the models are a simplification of the real-world setting. The results are limited by the availability of disease incidence estimates from robust surveillance systems and therefore may not reflect the most up-to-date burden of disease in Italy. Future studies should update these assumptions in the model to reflect the changing serotype dynamics. 

Furthermore, as only limited data are available on some parameters, we had to make certain assumptions. For example, AOM incidence from 2010 to present was assumed to be stable due to the lack of evidence on the change in mucosal disease following the implementation of PCV13 and some regional data such as pneumonia-related hospitalizations or serotype epidemiology were assumed to be representative of the national picture as no potential bias was knowingly introduced. Our results also represent an underestimation of the full value of pediatric vaccination as we did not include the broader impact vaccination has on educational attainment, anti-microbial resistance reduction, and increased productivity [26,27]. 

In conclusion, this modeling analysis shows the public health and economic benefits of maintaining the PCV13 strategy in Italy compared with switching to PCV10. Switching from PCV13 to PCV10 would increase the total incidence of disease linked to the re-emergence of serotypes 3 and 19A in the infants and elderly. The PCV13 strategy proved be a cost-effective approach in almost all scenarios considered, applying variations of parameters and model assumptions in a previously validated model. The use of this forecasting model greatly simplifies a very complicated picture, allowing the evaluation of historical and projected data and the analysis of changes in incidence over time.

## 4. Materials and Methods 

### 4.1. Model Design

A decision-analytic forecasting model was applied to estimate the public health and economic impact of changing the vaccination program switching from PCV13 to PCV10 compared to the maintenance of PCV13 in terms of incidence of invasive and non-invasive pneumococcal diseases covered by vaccines and direct medical costs, in the Italian context. This model has been described in detail elsewhere [16,17].

### 4.2. Model Assumptions

The model used real-world surveillance data over an observed, historical period to forecast serotype distribution and disease incidence over a specified 5-year time horizon. The population was stratified into seven age-groups (0–2, 3–4, 5–17, 18–34, 35–49, 50–64, and 65+ years). All serotypes contained within PCV13 and PCV10 were separately modelled by age group based on historical surveillance data.

The identified historical trends were used to predict future serotype behaviour under either PCV10 or PCV13 vaccine pressure, by age group. For example, in a country like Italy using PCV13, the observed data for the 13 serotypes contained within the vaccines were entered into the model to estimate historical disease trends for covered serotypes. The remaining non-PCV13 serotypes were grouped and modelled as non-covered disease across the same age groups outlined above, since neither vaccine covers these serotypes. PCV10 was forecasted based on the experience seen in Finland [28].

Italy first introduced a PCV7 program in 2007, which was replaced by PCV13 in 2010; both programs were implemented using a 2 + 1 schedule.

In the current analysis, we evaluated the clinical and economic impact of switching from PCV13 to PCV10 in Italy, assuming 89% [29] of infants were vaccinated. The remaining individuals of 0–2 years constitute the non-vaccinated cohort. Since we assumed that Italy would continue to follow a 2 + 1 schedule for PCV13 vaccine, PCV10 was forecasted given the experience seen in Finland [28], a country with a PCV10 strategy following a 2 + 1 schedule. 

Given the country and serotype-specific forecasts, the number of cases of IPD, pneumococcal pneumonia, and pneumococcal acute otitis media (AOM) were estimated. Rates of pneumonia and AOM were calculated based on all-cause disease incidence with an assumed proportion of 20% of disease due to pneumococcus [15,30].

Due to the limited real-world effectiveness data on the impact of PCVs against vaccine-type non-invasive pneumococcal diseases, the rates of mucosal disease were assumed to change proportionally related to the same serotypes causing IPD.

In the base case analysis, indirect effects in unvaccinated subjects were only included for IPD, given the serotype trend lines inherently capture disease behaviour in both vaccinated and unvaccinated age groups.

### 4.3. Epidemiologic Setting and Inputs

Serotype-specific IPD incidence data in under 5-year-olds was derived from the literature [15], which reported IPD incidence trends from 2008 to 2014 based on the national surveillance system. Total IPD incidence for over 5-year-olds was obtained from estimates across seven regions in Italy from 2008–2014, representing 43% of the national population [31].

We applied the serotype distribution in under 5-year-olds to the IPD incidence rates in over 5-year-olds to obtain serotype-specific IPD, as data on the distribution of serotypes in the class over 5 years are not detected by the surveillance system. With limited data beyond 2014, we conservatively assumed the incidence and serotype distribution remained flat through 2016; however, assumptions beyond this timeframe may not accurately capture the serotype dynamics in Italy, which require on-going surveillance.

We assumed the proportion of IPD caused by meningitis to be consistent with previously published estimates [32]. At the year of modelled switch, the incidence of IPD among 0–2-year-olds was 2.98 per 100,000 with 55% of remaining disease due to non-PCV13 type disease (Figure 1). 

The steep trend for >65-year-olds may be explained by the current serotype distribution in Italy and the trends observed in Finland (Figure 1). In Italy, a large proportion of remaining disease is due to non-vaccine type disease and in Finland, under a PCV10 program non-vaccine type disease has been increasing. Therefore, when we apply the PCV10 trend lines from Finland to Italy, there is a steep incline in non-vaccine type disease. Across all ages, 2.6 cases of IPD per 100,000 remained with 53% due to non-PCV13 type disease and 8–10% each due to serotypes 1 and 14 [15].

The incidence of mild AOM from 2007–2010 was obtained from the literature [33], and the incidence of hospitalized AOM was obtained from adjusting the proportion of hospitalized to non-hospitalized AOM [34]. AOM incidence from 2010–present was assumed to be stable due to the lack of evidence in Italy on the change in mucosal disease following the implementation of PCV13. The incidence of hospitalized pneumonia was obtained from a study of pneumonia-related hospitalization in the Veneto region [35] for adults ≥18 years and assumed to be representative of national data. The incidence of hospitalized pneumonia in children under 18 years was obtained from a syndromic surveillance system active in the emergency departments of the main hospitals of the Liguria region [36]. We estimated non-hospitalized cases for all ages by assuming 60% of pneumonia cases were hospitalized [37]. *S. pneumoniae* was assumed to cause 20% of pneumonia and 20% of AOM [30,31]. Case fatality rates for IPD were derived from the literature for children <5 years old [38].

In the base case analyses, cases of disease, deaths, and associated costs and quality-adjusted life years (QALYs) were estimated assuming PCV13 was maintained in the National Immunization Program (NIP), compared with a scenario where PCV13 was replaced with PCV10. We assumed indirect effects for invasive disease for both vaccines based on observed historical trends in non-vaccinated cohorts. In a switch from PCV13 to PCV10, we assumed a 1-year lag in disease trends among individuals over 5 years old due to the residual protection from the three additional serotypes.

### 4.4. Economic Input

Calculated costs and outcomes for the vaccination strategy included the number of disease cases and deaths avoided, QALYs gained, and total costs from a payer perspective. Costs and outcomes for each PCV were estimated in accordance with the WHO- CHOosing Interventions that are Cost-Effective (CHOICE) [39,40]. PCVs were cost-effective if they averted one QALY for less than three times per capita gross domestic product (GDP) and highly cost-effective at less than one times GDP per capita.

Direct medical costs associated with IPD, pneumonia, and AOM in Italy were obtained from national databases [41] and from the literature [42,43] (Table 3). No indirect costs were included in the analysis.

The cost of PCV13 and PCV10 were calculated based on a €20 difference. A €5 administration fee was included for both vaccines.

An incremental cost-effectiveness ratio (ICER) was calculated to estimate the cost-effectiveness of changing vaccination strategies. ICER, World Health Organization (WHO) and National Institute for Health and Care Excellence (NICE) cost-effectiveness thresholds were estimated to be €44,500 ($ 50,000), €31,953 (Italian GPD pro capita) and €25,600 (£20,000, using the pre-Brexit exchange rate) [40,45]. The ICER is calculated as the incremental change in costs divided by the incremental change in health outcome, and indeed, is generally described as additional cost per additional health outcome. An incremental analysis is used to examine the additional costs and additional effects of one vaccine with respect to another. [46].

Costs and outcomes were discounted at an assumed rate of 3.0% for Italy [47].

The study was conducted from the perspectives of the third-party payer (National Health Service).

### 4.5. Utilities

Utility decrements were applied for each occurrence of disease relative to an age-specific baseline utility weight [48] for individuals who did not experience a case of disease. Annual decrements of 0.0079 and 0.0232 were assumed for bacteremia and meningitis [49], respectively, and decrements of 0.0050, 0.0040, and 0.0060 were assumed for AOM, inpatient pneumonia, and outpatient pneumonia [32,50]. Sequelae such as neurologic impairment and hearing loss following a case of meningitis were assumed to occur with a probability of 25% and 2% [51], respectively, and carried a lifetime QALY decrement of 0.40 and 0.20 [48,52].

### 4.6. Analysis

Several scenarios and sensitivity analyses were undertaken to validate the robustness of results. Scenarios were run varying the time horizon, including indirect effects due to hospitalized pneumonia, and the impact of both vaccines, and PCV13 alone, on AOM due to non-typeable *H. influenzae* (NTHi) and *Moraxella catarrhalis*. Additional scenarios were evaluated using PCV10 serotype trends from the Netherlands, which implemented PCV10 in 2011 in a 2 + 1 schedule [53]. Given the natural variation in vaccine implementation and uptake between countries, the scenario using PCV10 trends from the Netherlands presents a potential incidence trajectory based on vaccine pressure in a different environment.

We included an impact of PCVs against all (single-species and co-colonized episodes) NTHi and *M. catarrhalis* AOM. This assumption is based on recent studies indicating that PCV13 may have a broader impact on non-pneumococcal AOM due to averting early onset cases, thereby averting later onset of more complex cases, often caused by non-pneumococcal pathogens [54]. This was accomplished by applying an annual rate of change in disease for PCV13 of 0.755 (NTHi) and 0.759 (*M. catarrhalis*) against the residual AOM caused by each pathogen. Due to more limited evidence on the impact of PCV10 against non-pneumococcal AOM, an annual rate of change in disease for PCV10 of 0.785 [55] (NTHi) and 0.0 (*M. catarrhalis*) was applied [56]. We estimated NTHi and *M. catarrhalis* to cause 31.7% and 1.6% of AOM disease in Italy [56]. 

We also tested the robustness of input parameters in a one-way sensitivity analysis. Individual parameters and assumptions varied based on 95% confidence intervals (CIs) or ±20%. A probabilistic sensitivity analysis was also performed (second-order Monte Carlo simulations) over 5000 random draws (Supplementary Figures S1 and S2). The proportion of IPD that is meningitis, vaccination rate, the percentage of all-cause OM that is pneumococcal OM, the percentage of all-cause pneumonia that is pneumococcal, utilities, and disease-specific case fatality rates were drawn from beta distributions. Limits on forecasted incidence, pre-vaccine incidence, direct costs, vaccine acquisition costs, vaccine administration costs, sequelae costs, lost productivity, and all-cause mortality rates were drawn from gamma distributions.

## Figures and Tables

**Table 1:** Incremental cases, deaths, and costs under a PCV13 versus PCV10 vaccination program, over a 5-year time horizon.

Parameter	Italy		
	PCV13	PCV10	Incremental
Morbidity			
IPD cases	7168	12,659	−5491
Acute otitis media cases	555,730	949,633	−393,902
Pneumonia cases	427,623	453,855	−26,233
Total cases	990,520	1,416,147	−425,627
Mortality			
IPD cases	1061	1875	−814
Outcomes			
QALYs gained	211,218,952	211,215,677	3275
Direct medical cost			
Vaccination program cost	€461,385,903	€280,093,471	€181,292,432
IPD	€35,894,686	€63,247,183	−€27,352,496
AOM	€44,396,639	€72,265,426	−€27,868,787
Pneumonia	€723,483,604	€754,708,583	−€27,868,787
Total cost	€1,265,160,833	€1,170,314,663	€94,846,170
Incremental cost-effectiveness			
Cost per QALY gained			€28,963 PCV13 cost-effective

Invasive pneumococcal disease, IPD; acute otitis media, AOM; quality-adjusted life-year, QALY.

**Table 2:** Scenario analyses and the incremental costs and (QALY) under a PCV13 versus PCV10 vaccination program.

	PCV13		PCV10		Incremental	
Scenario	Cost	QALYs	Cost	QALYs	Cost	QALYs
Base case	€1,265,160,833	211,218,952	€1,170,314,663	211,215,677	€94,846,170	3275
Including NTHi and *Moraxella catarrhalis* for both vaccines	€1,337,750,244	211,216,821	€1,253,389,766	211,213,238	€84,360,478	3583
Excluding NTHi and *M. catarrhalis* for PCV10	€1,337,750,244	211,216,821	€1,323,200,530	211,211,189	€14,549,714	5632
Ten-year time horizon	€2,180,992,438	364,913,596	€2,034,702,338	364,904,743	€146,290,100	8853
Netherlands PCV10 trend line	€1,266,772,485	211,218,959	€1,105,206,477	211,218,306	€161,566,008	653
Including indirect effects on hospitalized pneumonia	€1,216,099,224	211,219,055	€1,164,320,376	211,219,055	€51,778,848	3360

Quality-adjusted life years, QALY; Non-typeable H. influenza, NTHi.

**Table 3:** Epidemiological inputs used in the cost-effectiveness analysis of infant pneumococcal vaccination program.

Input	Age Range (years)							Source
	<2	2–4	5–17	18–34	35–49	50–64	≥65	
Population	1,492,050	1,080,898	7,433,899	11,252,659	14,290,635	12,066,427	13,014,942	[44]
Disease rates in 2016 (per 100,000 person-years)								
Invasive pneumococcal disease (IPD)	2.98	2.98	0.99	0.36	1.46	1.43	7.7	[21,23]
Case fatality rate (CFR)	0.15	0.15	0.15	0.15	0.15	0.15	0.15	[15]
Pneumococcal meningitis								
Incidence	0.61	0.15	0.18	0.03	0.15	0.12	0.18	[15]
Case fatality rate (CFR)	0.15	0.15	0.15	0.15	0.15	0.15	0.15	[15]
Hearing loss, probability of (%)	0.2							[40]
Neurological sequelae, probability of (%)	0.25							[40]
Inpatient pneumonia								
Incidence (per 100,000 person-years)	2190.9	2190.9	191.4	51	51	51	1,280	[26,27,29]
Outpatient pneumonia								
Incidence (per 100,000 person-years)	1079.1	1079.1	95.7	34	34	34	853	
Simple AOM								
Incidence (per 100,000 person-years)	19300	19700						[25]
Direct medical costs (local currency)								
Pneumococcal bacteremia	3176	3176	3176	5493	5493	5493	5493	[32,33,34]
Pneumococcal meningitis	8067	8067	8067	8067	8067	8067	8067	[32,33,34]
Pneumonia inpatient	2190	2190	191.4	51	51	51	1280	[32,33,34]
Pneumonia outpatient	1079	1079	95.7	34	34	34	853.3	[32,33,34]
Simple AOM	76	76	76					[32,33,34]

PCV10, 10-valent pneumococcal conjugate vaccine; PCV13, 13 valent pneumococcal conjugate vaccine; AOM, acute otitis media.

## Supplementary Materials

The following are available online at https://www.mdpi.com/2076-0817/9/2/76/s1: Figure S1, One-way sensitivity analysis; Figure S2, Probabilistic sensitivity analysis; Table S1, Total incremental diseases cases and deaths avoided with a PCV13 program, vaccination costs, and budget impact by Italian Region.
