# Supplementary material for: Estimating the Clinical and Economic Impact of Switching from the 13-Valent Pneumococcal Conjugate Vaccine (PCV13) to the 10-Valent Pneumococcal Conjugate Vaccine (PCV10) in Italy
Source: Pathogens. 2020 Jan 22;9(2):76. doi: 10.3390/pathogens9020076 (PMC7168640; doi:10.3390/pathogens9020076)
Supplement: Supplementary file 1 [file pathogens-09-00076-s001.pdf]

Supplementary figure S1

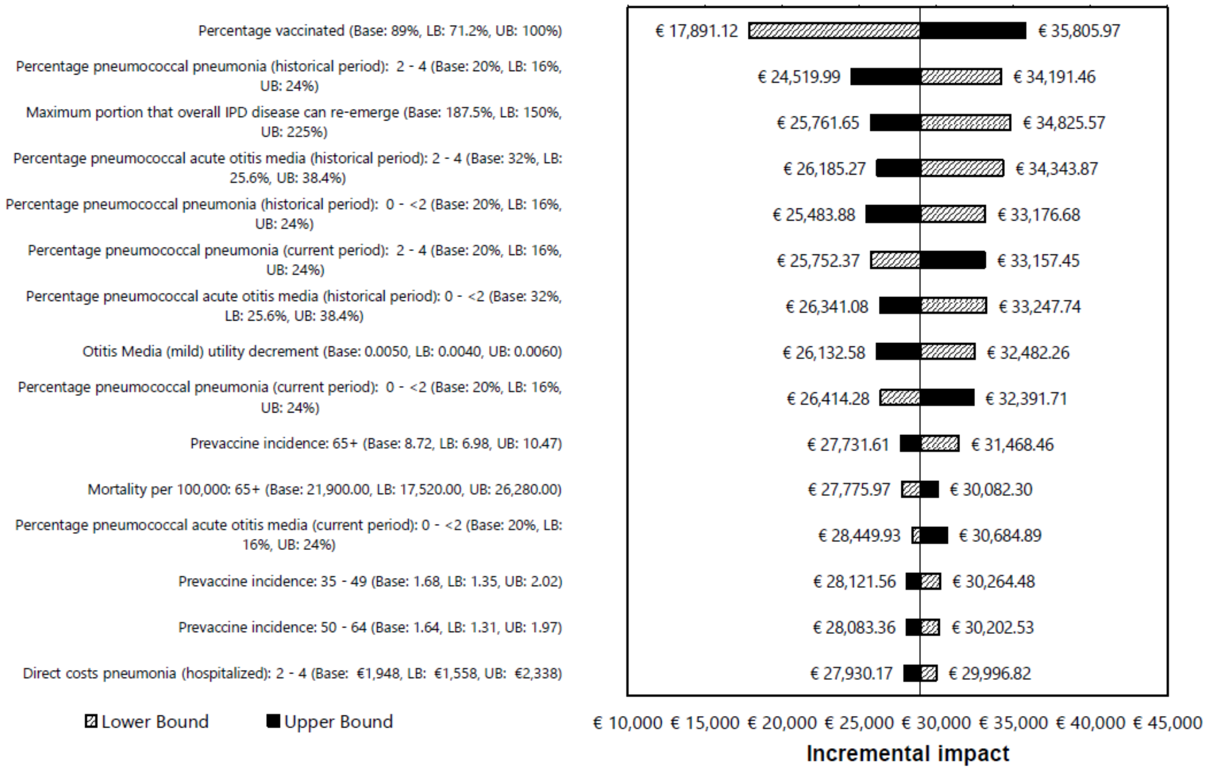

Supplementary figure S2

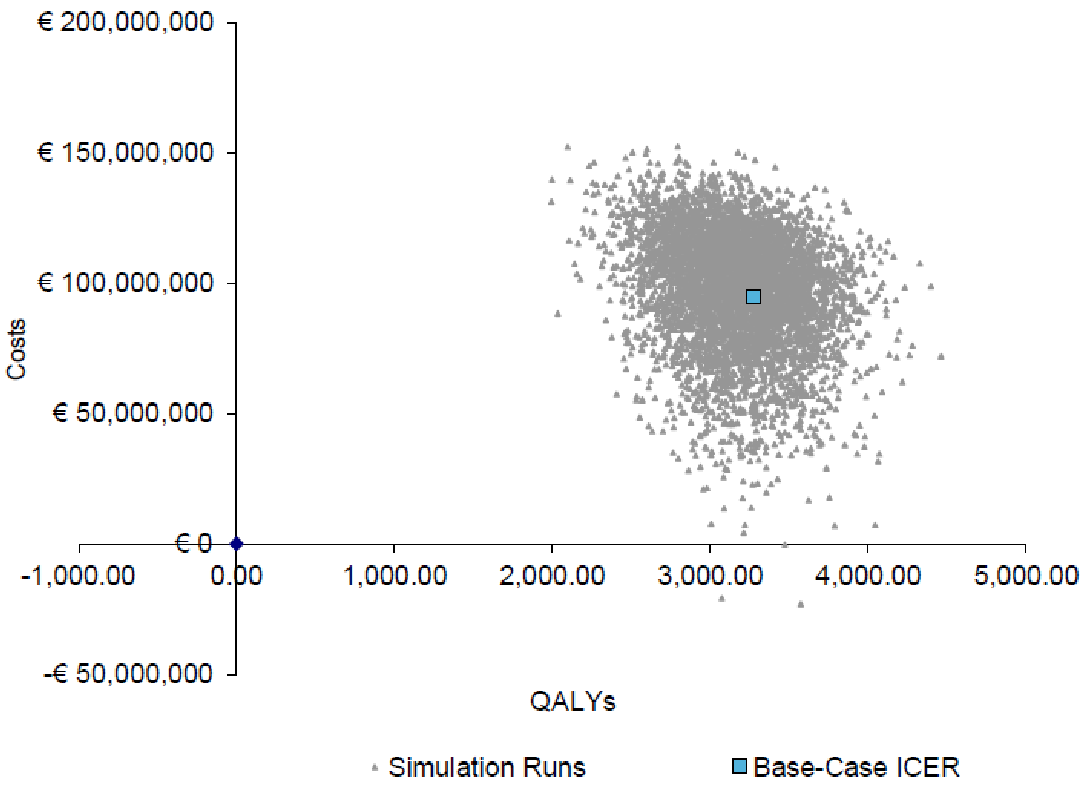

Supplementary Table S1

Total incremental diseases cases and death avoided with a PCV13 program, vaccination costs, and budget impact by Italian Region.

Cumulative over 5 Years (PCV13 vs PCV10)	Incremental Disease Cases Avoided (PCV13 vs PCV10)	Incremental Deaths Avoided (PCV13 vs PCV10)
Abruzzo	8,543.0	16.3
Basilicata	4,018.2	7.7
Calabria	13,844.3	26.4
Campania	41,136.4	78.4
Emilia Romagna	31,342.1	59.7
Friuli Venezia Giulia	8,579.9	16.4
Lazio	41,552.3	79.2
Marche	10,835.6	20.7
Liguria	11,027.6	21.0
Lombardia	70,585.1	134.5
Molise	2,187.1	4.2
Piemonte	30,945.4	59.0
Puglia	28,630.1	54.6
Sardegna	11,646.3	22.2
Sicilia	35,624.1	67.9
Trentino Alto Adige	7,487.9	14.3
Tuscany	26,365.5	50.3
Umbria	6,262.4	11.9
Valle d'Aosta	893.9	1.7
Veneto	34,573.6	65.9
